# Πάντα ῥεῖ καὶ οὐδὲν μένει^1^

**DOI:** 10.3201/eid1210.AC1210

**Published:** 2006-10

**Authors:** Polyxeni Potter

**Affiliations:** * Centers for Disease Control and Prevention, Atlanta, Georgia, USA

**Keywords:** Pierre-Auguste Renoir, Renoir, Boating Party, Cover Story, Everything Flows, Nothing Stands Still

**Figure:**
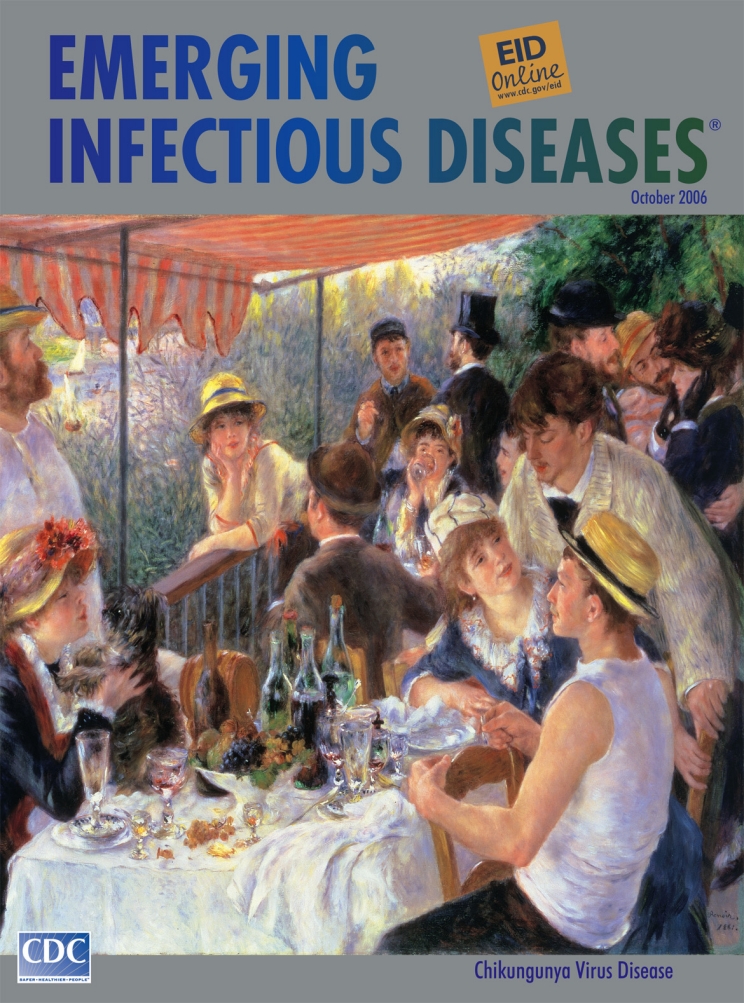
**Pierre-Auguste Renoir (1841–1919).** Luncheon of the Boating Party (1880–1881). Oil on canvas (130.175 cm × 175.5775 cm). Acquired 1923. The Phillips Collection, Washington, DC, USA

– Herakleitos of Ephesus, c. 500 bc

"I'm doing a painting of oarsmen which I've been itching to do for a long time. I'm not getting any younger, and I didn't want to defer this little festivity which later on I won't any longer be able to afford already it's very difficult…; one must from time to time attempt things that are beyond one's capacity," wrote Pierre-Auguste Renoir to Paul Bérard, his friend and supporter (*1*). The ambitious painting might have been Renoir's response to author and critic Émile Zola's challenge to impressionists in 1880 that, instead of "sketches that are hardly dry," they "create complex paintings of modern life" after "long and thoughtful preparation" (*2*).

Zola was not alone in challenging artists of his generation. Poet Charles Baudelaire back in 1863 had called for a "painter of modern life," inviting his contemporaries to create art of their own times (*3*). Heeding the call, French artists of the late 19th century broke with the past, forging perhaps the most popular movement in the history of art. Radical in their departure from tradition, the impressionists abandoned history as source of inspiration and moved the studio outdoors to capture a moment, an impression, under the changing light of the sun. No longer interested in telling a story, they replaced standard narrative techniques with feathery, interrupted brush strokes that best described an angle of interest or a fleeting scene of daily life. Their innovations forever changed how art was created and viewed, producing works of unprecedented spontaneity and lightness.

The child of working-class parents, Renoir was born in Limoges, the city in France known for its porcelain and fine china. When he was still young, the family moved to Paris and settled in the Louvre area, where he grew up lighthearted and easygoing in the shadow of the great museum. Despite early affinity for scribbling and drawing, his fine singing voice was noticed first, and he studied under composer Charles Gounod, who encouraged a music career for him. Because of financial constraints, at age 13, he was apprenticed instead to a porcelain painter with the prospect of long-term work at a large factory outside Paris.

At the porcelain shop, his talent for painting was quickly acknowledged, but he continued to paint china, fans, café murals, and window shades, to study in the evenings, and to copy the masters at the Louvre until he could enroll in the École des Beaux-Arts. There, he met Claude Monet (*4*), Frédéric Bazille, and Alfred Sisley. The foursome, who shared an aversion to established rules, bonded quickly, painted together, influenced each other, and became founding members of impressionism, along with Paul Cézanne, Edgar Degas, Berthe Morisot, and Camille Pissarro.

"There are no poor people" Renoir believed, denying that lack of means could interfere with happiness, success, or the imagination (*5*), even though living expenses and painting supplies were hard to come by for much of his career: "I would several times have given up if Monet had not reassured me with a slap on the back" (*5*). And when near the end of his life he was invited to the Louvre to view the hanging of one of his works, he mused, "If I had been presented at the Louvre 30 years ago in a wheelchair I would have been shown the door" (*5*).

Many innovations of this era, among them mixed paints in metal tubes and use of poppy seed oil as binding medium, facilitated the artists' task. And the advent of photography freed them from the need to paint in realistic detail, proposing new ways to focus on subjects. But turmoil brought on by the Franco-Prussian war in 1870 interfered with the full impact of these changes as with the progress of impressionism. Bazille was killed in action; Monet, Pissarro, and Sisley moved to England; and Renoir joined the cuirassiers, though he saw no action.

After the hostilities, Renoir returned to Paris, where he continued to struggle for acceptance. He worked with Monet, painting on the banks of the Seine and the coast of Normandy; traveled to Africa, Spain, and Italy; and by the end of the decade, he had found his own unique voice. His style became more refined and traditional, focusing closely on the human form. "For my part," he freely admitted, "I always defended myself against the charge of being a revolutionary. I always believed and I still believe that I only continued to do what others had done a great deal better before me" (*5*).

They are "lumpy and obnoxious creatures," wrote the New York Sun about Renoir's famed female nudes, exhibited in New York in 1886 (*5*). Nonetheless, popular success and creature comforts were finally within the artist's reach. He remained extremely prolific (more than 6,000 paintings); despite debilitating arthritis and other health problems, he continued to paint until his death.

No other painting captures Renoir's characteristic joie de vivre and conviviality better than Luncheon of the Boating Party on this month's cover. The social ease and camaraderie of his youth seem to have permeated this painting, while gaiety and charm emanate from it toward the viewer, who is tempted to join in. "The picture of rowers by Renoir looks very well," wrote Eugene Manet to his wife Berthe Morisot (*6*). Holiday-making was a favorite theme of the impressionists, who "…show their particular talent and attain the summit of their art when they paint our French Sundays…kisses in the sun, picnics, complete rest, not a thought about work, unashamed relaxation" (*6*).

Renoir worked on the complex composition for months, frustrated at times with the unavailability of models, the clustering of figures, the landscape: "…I no longer know where I am with it, except that it is annoying me more and more" (*1*). On this single canvas, he combined still life, genre, landscape, and portraiture to capture food, friends, and conversation near the waterfront. Carefully structured and meticulously finished, this moment at play was to become a cultural icon.

The gathering took place near Chatou, Renoir's favored retreat on the Seine. Once the domain of the affluent, the area now offered pastimes for all. A group of friends assembled on the balcony of the Maison Fournaise. Among them, a historian and art collector, a baron, a poet and critic, a bureaucrat, actresses, and artists Paul Lhote and Gustave Caillebotte, who sat backward in his chair in the right foreground and gazed across the table at Aline Charigot, the young seamstress who later would become Renoir's wife. The youths leaning against the rail are proprietors of the establishment (*7*).

"…[O]ne cannot imagine these women…having been painted by anybody else," wrote art critic Théodore Duret, "They have…that graciousness, that roguish charm, which Renoir alone could give to women" (*8*). Earthy, savvy, and engaging, they light up the scene. The luncheon is finished. The crowd "hangs out" against the clutter of leftover food and drink, gracing the intimate tableau all of us want to be in.

Just beyond the awning, the river flows discreetly in the background. Soon, it will turn dark, the crowd will disperse, the moment will end. The moment and its transient place in constant change, so well understood by the impressionists and masterfully captured by Renoir, have also long puzzled philosophers and scientists and are central to the study of emerging disease. In a world where "everything flows," organisms and their surroundings are constantly changing, and "nothing stands still," vigilance is order of the day. Disease control is as good as the next set of natural circumstances, for as Herakleitos of Ephesus put it 2,500 years ago, "You cannot step twice into the same river, for fresh waters are ever flowing upon you" (*9*).
